# A Narrative Review of New Treatment Options for Diabetic Nephropathy

**DOI:** 10.7759/cureus.33235

**Published:** 2023-01-01

**Authors:** Aadhira Pillai, Darshna Fulmali

**Affiliations:** 1 Physiology and Biochemistry, Jawaharlal Nehru Medical College, Datta Meghe Institute of Medical Sciences, Wardha, IND; 2 Anatomy, Jawaharlal Nehru Medical College, Datta Meghe Institute of Medical Sciences, Wardha, IND

**Keywords:** fibrotic, mechanism, prognostic markers, diagnostic efficacy, ’ ‘treatment options for diabetic nephropathy

## Abstract

Diabetic nephropathy (DN) is a type of nephropathy that is caused by a diabetic condition. Diabetic nephropathy is seen in type 1 and type 2 diabetes. End-stage renal disorders are brought on by DN. Diabetic nephropathy is thought to be linked to metabolic changes in the body. Proteinuria and glomerular filtration rate are the two most crucial diagnostic and prognosis measures for diabetic kidney disease (DKD), yet both have significant disadvantages. Novel biomarkers are thus increasingly required to improve risk factors and detect disease at an early stage. Controlling blood glucose and vital sign like body temperature and blood pressure, reducing cholesterol levels, and blocking the renin-angiotensin system are the standard treatments for diabetic patients. On the other hand, if used too late within the course of the disease, these therapeutic techniques can only provide partial relief from nephropathy.

The complicated pathophysiology of the diabetic kidney, which experiences a variety of severe structural, metabolic, and functional alterations, represents one of the most important obstacles to the event of effective therapeutics for DN. Despite these issues, new diabetes models have identified promising treatment targets by identifying the mechanisms that control important functions of podocytes and glomerular endothelial cells. It has been shown in the vast majority of trials that renin-angiotensin system inhibitors combined with integrative therapies work well for DN. Combining sodium-glucose cotransporter-2 inhibitors and renin-angiotensin-aldosterone system blockers is a novel way to slow down the course of DKD by lowering inflammatory and fibrotic indicators brought on by hyperglycemia, which is more effective than using either medicine alone. Aldosterone receptor inhibitors and advanced glycation end-product inhibitors are two recently produced medications that may be used successfully to treat DN.

## Introduction and background

Diabetic nephropathy could be a type of nephropathy caused by a diabetic condition. Diabetic nephropathy (DN) may be a common complication generally seen together in type 1 and type 2 diabetes [1]. This can be the foremost common complication for proteinuric and non-proteinuric end-stage renal disease (ESRD) [2]. This condition eventually ends up in diabetic kidney disease (DKD) which may be a leading reason for morbidity in chronic nephropathy which is chronic kidney disease (CKD) [3]. A collection of physiological dysfunctions known as diabetes mellitus (DM) is characterized by persistent hyperglycemia. A xanthophyll carotenoid called astaxanthin can reduce blood sugar levels by maintaining beta-cell activity, enhancing insulin resistance (IR), and boosting insulin secretion [4]. The maintenance of the kidney is important as it regulates the other functions of the body: Removal of waste products from the body, maintaining body fluid levels, balancing body blood pressure, maintaining healthy bones, and helping produce red blood cells.

Renal disease is an indication that the kidneys; normal functions have been compromised. Kidney damage from diabetes can be severe. Once injured, the kidneys are unable to perform any regular tasks, including blood filtration. Hence as a result of kidney failure, the body fills up with water and waste materials. Uremia is the name for this illness. In this circumstance, pedal edema will be seen. Additionally, the body needs clean blood to operate properly, the patient will feel exhausted and weak. Clinical manifestations seen in diabetic nephropathy are advanced albuminuria, a decrease in projected glomerular filtration rate, and renal diseases. Hyperglycemia-induced metabolic alterations that result in glomerular hypertrophy, glomerulosclerosis, tubulointerstitial inflammation, and fibrosis are hypothesized to be the etiology of diabetic nephropathy [5]. Based on new studies, an intermediate phenotype combining stress and chronic inflammation may potentially occur, whereas pressure-interfering damage is a mechanism that is known for chronic kidney diseases. Chronic inflammation in CKD is brought on by several factors, including oxidative damage experienced by local renal cells. Regulation of proinflammatory and anti-inflammatory factors like nuclear factor, erythroid 2 related factor 2 (Nrf2), and nuclear factor (NF)-B-mediated gene transcription are crucial for the response of glomerular and tubular cells to kidney injury. Chronic inflammation impacts chronic kidney patients with declining glomerular filtration rate (GFR). The pathophysiology of the evolution of obesity-related kidney injury involves an important energy-sensing switch known as adenosine 5′ monophosphate (AMP)-activated protein kinase (AMPK) [6].

The main structural abnormalities causing diabetic nephropathy in type 1 diabetes are the thickening of the glomerular basement membrane (GBM) and mesangial enlargement, but there are also arteriolar, tubular, and interstitial lesions. The systemic indicator of mesangial expansion in type 1 diabetes that coordinates with all renal functional measures is the mesangial fractional expansion. On the other hand, clinical research has shown that circulating Exo-miRs (exosomal microRNAs) that have been extracted from urine or serum have a lot of promise as promising biomarkers in DN. There are no known structural or functional links between type 2 diabetes. In this research, patients with type 1 and type 2 diabetes with early-onset nephropathy (microalbuminuria) problems have their renal structure examined. When the albumin excretion rate (AER) was greater than 30 micrograms per minute, both GBM widths were higher in type 1 diabetes patients in microalbuminuria circumstances compared to normoalbuminuric (NA) individuals [5]. To determine if glomerular and interstitial lesions progress together, 11 type 1 diabetes patients underwent successive renal biopsies five years apart. While the glomerular filtration rate stayed stable over five years, AER increased considerably [6]. The initial state of all structural metrics was aberrant, with mean glomerular volume rapidly increasing while GBM width and the interstitial volume portion remained stable. Additionally, there was a correlation between the change and the change in AER (r = 0.64, p < 0.05). Thus, continued mesangial expansion is the key factor at the illness stage during which some individuals develop microalbuminuria (MA) or proteinuria, although additional interstitial expansion does take place. Additionally, a sizable number of type 2 cases were investigated. Microscopy was used to identify early diabetic glomerulopathy in NA individuals and was found to be more advanced in MA and proteinuria patients [7]. Through microscopy that was similar to those obtained using light microscopy. In contrast, 40% of MA patients had more severe tubulointerstitial and/or vascular lesions, 30% of patients had normal renal structure and only 30% of MA patients had the characteristic diabetic glomerulopathy. The goal of the article is to give a brief idea about the treatment that can be used to treat diabetic nephropathy.

## Review

Diagnostic/prognostic biomarkers of diabetic kidney disease

End-stage renal illness is most commonly attributed to DKD which leads to ESRD. The most important diagnostic and prognostic indicators for DKD are albuminuria and glomerular filtration rate and both have considerable drawbacks. Novel biomarkers are thus increasingly required to improve risk factors and detect disease at an early stage [8]. The main shortcomings of the currently accessible indicators will be outlined, along with intriguing novel biomarkers and their prospective clinical applications, in this study. This article underlines the role that recent multi-omic technology developments have played in identifying new DKD biomarkers [3]. Also, this article updates its readers on recent, cutting-edge techniques for examining renal shape and function using imaging and functional tests. Damaged organelles and macromolecules are recycled and softened by the conserved multistep autophagy system to maintain intracellular homeostasis. Autophagy plays a significant role in several diseases, particularly renal ailments. According to certain studies, glomerular and tubulointerstitial kidney illnesses in diabetic individuals are related to dysregulated autophagy. As a result of these advances in our understanding of autophagy in DN, we have to identify a replacement therapeutic target for the prevention and treatment of diabetic nephropathy. A progressive microvascular diabetes consequence is DN. A growing body of research demonstrates that chronic mitochondrial dysfunction, which disrupts mitochondrial homeostasis and consequently impairs healthy kidney function, promotes the event of renal disorders, including DN.

This article reviews pharmacological control of mitochondrial networking as a possible treatment approach for stopping and treating DN and the current developments in understanding mitochondrial networking and signaling pathways in healthy and pathological situations. The potential benefit of alternative treatments for DN, including herbal medicine and lifestyle modifications, has also emphasized the biological underpinnings of non-traditional treatments for mitochondrial dysfunction and their potential contributions [9]. Future research will benefit from the emergence of novel understandings via mitochondrial networking. Long non-coding RNAs (lncRNAs), which play important roles during a big selection of biological activities, are revealed by recent developments in large-scale RNA sequencing and genome-wide profiling initiatives significant to notice that abnormal lncRNA expression has been linked to many human diseases including several kidney disorders. These findings have increased the likelihood that lncRNAs might be untapped therapeutic targets for renal disorders. However, there are many significant concerns about how lncRNAs function and the way they relate to kidney illnesses that require to be properly addressed [10]. The methods of action of lncRNAs and their promise as therapeutic targets in kidney diseases, with a focus on the roles of best-characterized lncRNAs, implicated in the pathogenesis and progression of diabetic nephropathy. First off, one possible mechanism for AMPK protection against renal injury is its inhibitory effect on ferroptosis. Second, AMPK has a complicated impact on lipolysis since it both stimulates and inhibits basal lipolysis. Thirdly, through activating AMPK, statins may have renoprotective effects. Fourthly, type 2 diabetes has been linked to DN by certain microRNAs that target AMPK mRNA. Wide-ranging intracellular and external cellular interactions in various disorders have been associated with the Nrf2 gene as a molecular actor. Some transcription factors that are responsible for generating specific proteins involved in the regulation of metabolic and detoxifying enzymes are given by the regulation of this gene and contain antioxidant response elements (ARE) in their promoter regions [11]. DKD progression and prognosis are known to be accelerated by endothelial nitric oxide synthase (eNOS) dysfunction. One way this is done is because kidney injury is encouraged by hypercoagulability, which is linked to low eNOS levels. By activating the protease-activated receptors (PARs) in the extrinsic coagulation cascade, tissue factor (factor III) and downstream coagulation factors, such as active factor X (FXa), worsen inflammation [12].

Complications of DKD: Fluid retention, high force per unit area, may cause swelling in your arms and legs, stroke can result from cardiovascular conditions, injuries to the blood vessels in the light-sensitive tissue behind the line of sight (diabetic retinopathy), less red blood cells are needed to transport oxygen (anemia), foot ulcers, sexual dysfunction in men, diarrhea, and other issues related to harmed blood vessels and nerves, bone and mineral problems caused by the kidney’s inability to maintain the right level of calcium and phosphorus in the blood. The end-stage renal disease causes irreversible kidney deterioration, necessitating dialysis or a kidney transplant for survival.

Treatment for diabetic kidney disease

Controlling blood glucose and vital signs, reducing cholesterol levels, and blocking the renin-angiotensin system are the standard treatments for diabetic patients. On the other hand, if used too late within the course of the disease, these therapeutic techniques can only provide partial relief from nephritis. The complicated pathobiology of the diabetic kidney, which experiences a variety of severe structural, metabolic, and functional alterations, represents one of the most important obstacles to the event of effective therapeutics for DN. Despite these issues, new diabetes models have identified promising treatment targets by identifying the mechanisms that control important functions of podocytes and glomerular endothelial cells [13]. In this review, we will cover current developments in the area, examine important molecular pathways that are involved in the pathophysiology of the disease, and explain how these pathways might be altered to prevent or reverse DN. Diabetic nephropathy is one of the leading causes of death in people with type 1 and type 2 diabetes (DN).

According to current studies, the development and progression of DN are directly correlated with autoimmunity, specifically the autoantibodies that are essential to the pathogenesis of DN. G-protein coupled receptor, pancreatic, and autoantibodies linked to endothelial cell injury have been shown to make up the majority of autoantibodies found in the blood of DN patients at this time. Within the specific context of diabetes, high glucose (HG) can cause the development of several autoantibodies. These autoantibodies can modify the course of DN and mediate the impairment of renal function through several afferent routes. Therefore, it is critical to understand how autoantibodies influence the DN event.

Therapeutic advances

However, the vast majority of studies have demonstrated that renin-angiotensin system inhibitors in conjunction with integrative therapy are effective for treating diabetic nephropathy. The Renal Events in Diabetes with Established Nephropathy Clinical Evaluation (CREDENCE) trial, which presented a replacement treatment in 2019, demonstrated the effectiveness of a sodium-glucose cotransporter-2 (SGLT2) inhibitor against DKD. The challenges brought on by DKD have not yet been fully resolved. Patients who have suffered from hyperglycemia for a brief period can develop diabetes, including diabetic kidney damage, even after normal glucose levels. Epigenetic changes and accumulations of advanced glycation end products (AGE) act as the metabolic memory of transitory hyperglycemia. In the search for drugs that improve metabolic function, clinical and basic research mostly focused on AGE inhibitors and histone modification inhibitors. Additionally, incretin-related drugs have demonstrated nephroprotective effects in several clinical investigations; this research, whose main objective is kidney outcome, is still underway. Recently approved renal anemia medications that inhibit hypoxia-inducible factor prolyl hydroxylase may also be nephroprotective. Additionally, NF-E2-related factor 2 activators raised the glomerular filtration rate of DKD patients in the bardoxolone methyl treatment: Renal Function in Chronic Nephrosis and Sort 2 Diabetes (BEAM) trial and subsequently in the clinical trial Study of Bardoxolone Methyl in Patients with Chronic Nephropathy and Sort 2 Diabetes (TSUBAKI) trial. So, following the administration of an SGLT2 inhibitor, a variety of cutting-edge medicines may be employed to treat DKD [14]. 

Future study is expected to present novel viewpoints. As a result, more and more innovative medications are being developed. Examples include inhibitors of the SGLT2, glucagon-like peptide-1 (GLP-1), and dipeptidyl peptidase-4 (DPP-4) enzymes, all of which have good clinical efficacy [15]. Several recently developed medications for the treatment of DKD, such as protein kinase C (PKC) inhibitors, advanced glycation end product (AGE) inhibitors, aldosterone receptor inhibitors, endothelin receptor (ETR) inhibitors, transforming growth factor (TGF) inhibitors, Rho kinase (ROCK) inhibitors, and others, have shown promise in animal or clinical trials. We examined recent developments in DKD therapy, the state of some pharmacological research, and prospective new DKD treatment medications in this paper [16]. Combination target therapy for disorders including albuminuria, hyperglycemia, hypertension, and hyperlipidemia, among others, is essential for the whole management of DKD. The most current oral hypoglycemic medications, SGLT2 inhibitors, act by blocking SGLT2 in the renal proximal tubules, which reduces glucose absorption and increases urinary glucose excretion. They demonstrated remarkable results in direct renoprotective benefits and cardiovascular (CV) safety in addition to improvements in glycemic control by lowering albuminuria and the independent CV risk variables, such as body weight and blood pressure, among others [17]. By reducing the inflammatory and fibrotic markers brought on by hyperglycemia, combining SGLT-2 inhibitors with renin-angiotensin-aldosterone system (RAAS) blockers provide a unique strategy to slow the progression of DKD that is more successful than using either medication alone. The available animal and population-based research have characterized the SGLT2 inhibitors and RAAS blockers [18]. The innate immune molecule-targeting medications that are already on the market have been examined against DKD in clinical and preclinical settings, and they have discovered additional pharmacological targets that may eventually be used to treat DKD [19].

Most cell types secrete extracellular vesicles, also known as exosomes, through the invagination of the endosomal membrane. Exosomes are essential for mediating intercellular communication in both health and illness because of their ability to carry payloads like DNA, mRNA, proteins, and microRNA to nearby or distant cells. They can manage the recipient cells; biological activity because of this ability [20]. DN may be a devastating microvascular complication of diabetes and a huge financial burden on both individuals and society. It also significantly contributes to end-stage renal disease globally. There are no therapeutic methods that stop the advancement of DN despite repeated increased attempts, which suggests that new approaches are needed. According to several studies that suggest their involvement in pathophysiological diabetic nephropathy processes, exosomes may be potential indicators and therapeutic targets for diabetic nephropathy [21]. The most recent advancements in exosome processes in DN are outlined in this review because of their potential as markers and therapeutic targets. ESRD affects the kidney and renal tubules and may be a disabling side effect of both type 1 and type 2 diabetes. A large population is impacted because DN affects over 1/2 of all diabetic patients during their lifetime and diabetes could be a global epidemic. Due to the complexity of the disease, existing methods of diagnosis and therapy are unable to forestall disease progression or offer a reliable cure [22]. Today, most renal physiology is regulated by inflammatory chemicals, which makes DN a signal of inflammation. Numerous susceptibility genes connected with DN have been discovered recently because of advancements in genetics and genomic technologies, many of which have inflammatory properties. We will speak about the current state of biomarker and molecular therapy development for enhancing precision medicine supported by their involvement in DN.

Green tea has historically been used as an anti-diabetic drug and benefits diabetic nephropathy. These positive effects were thought to be caused by the tea’s hypoglycemic ability, which lowers the speed of advanced glycation end products and oxidative damage by reducing glycemic overload [23]. These findings, however, are still debatable because prior research has shown that tea always has the flexibility to provide a hypoglycemic effect. Advanced glycation end-products (AGEs) are heterogeneous compounds that are created when proteins, lipids, or nucleic acids are glycated during hyperglycemia without the use of an enzyme. A novel risk factor for the development of DN has been discovered: the buildup of AGEs in peripheral nerves [24]. The gold standard for AGE assessments in tissues is tissue biopsy. However, the assessment of those patients utilizing the more modern, speedy, and uncomplicated method of skin autofluorescence (SAF) has recently received interest due to its non-invasiveness, high repeatability, and acceptable association with the quality technique of skin biopsy. It has been proven that DN correlates on its own with the buildup of tissue AGEs as measured by SAF. Additional data demonstrates their potential value as early biomarkers of the latter, which is critical. Diabetes retinopathy, cardiovascular autonomic neuropathy, and diabetic nephropathy are further important connections. The effectiveness of skin AGEs for DN screening is still up for discussion [25].

Using cutting-edge technology, studies have identified several biomolecules that are related to the beginning of DN in T2DM. These biomolecules may have early DN biomarker potential [26]. Thrombin activates protease-activated receptors to carry out a variety of pathogenic actions (PARs). We predicted that albuminuria, which is a symptom of DKD progression, is correlated with reduced plasma heparin cofactor II (HCII) activity because HCII selectively inactivates thrombin [27]. The advancement of fibrosis and the inflammatory response are linked to 2-antiplasmin (2AP). Production of 2AP was driven by the high glucose levels, and 2AP is linked to EndoMT and the advancement of fibrosis in DN. These results offer a foundation for clinical approaches to enhance DN [28]. 

Discussion

**Figure 1:**
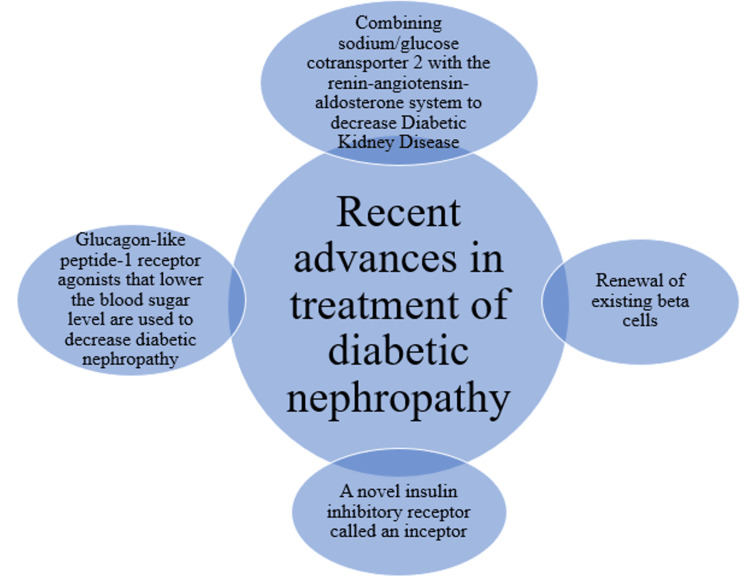
Recent advances in the treatment of diabetic nephropathy References [15,19]

This article reviews the currently available ways to partially delay the onset of DN. Further controlled functions of podocytes and glomerular endothelial cells lead to pathogenesis, and they are often modulated to stop or perhaps reverse DN. Also, different effective treatment was reviewed in this article for DN. The purpose of this is to critically review the most recent research on the relationship between skin AGEs and diabetic microvascular issues, with a focus on diabetic neuropathy, and to identify the most pressing knowledge gaps. RAAS lowering with angiotensin-converting enzyme inhibitors (ACEi) or angiotensin receptor blockers (ARBs) is the first-line treatment for the clinical management of DKD [29]. GLP-1 receptor agonists have been added to the treatment toolkit because of their well-established benefits for kidney protection and patient survival [30]. Evidence from cardiovascular outcome trials using SGLT-2 inhibitors strongly suggested that these drugs, in addition to standard-of-care therapy, significantly slowed the course of CKD in people with diabetes mellitus [31]. The finding that fructose can be created endogenously from glucose under pathologic circumstances, including diabetes, heart hypertrophy, and dehydration, is a recent achievement in science [32]. It is increasingly clear that histidine, one of the epigenetic pathways, acetylation (Ac), is hypothesized to be connected to diabetic retinopathy (DR), diabetic microangiopathy, and other vascular consequences of diabetes caused by DN and cardiomyopathy [33]. Modern-day diabetic human-specific lesions, such as nodular lesions and doughnut lesions, are what define nephropathy, exudative lesions, and lesions [34]. Current biochemical indicators, like the excretion of urine albumin, are used for early screening and monitoring of DN which has limitations (Figure 1).

## Conclusions

To avoid diabetic nephropathy (DN), diabetics must be regularly monitored. The most effective medications include those that block the renin-angiotensin system as well as newer hypoglycemic drugs such as sodium-glucose cotransporter-2 (SGLT2) and glucagon-like peptide-1 (GLP-1) inhibitors. The initial course of treatment for the clinical management of diabetic kidney disease (DKD) involves renin-angiotensinogen-aldosterone (RAAS) system lowering with angiotensin-converting enzyme inhibitors (ACEi) or angiotensin receptor blockers (ARBs). In addition, newly developed drugs such as advanced glycation end product (AGE) inhibitors and aldosterone receptor inhibitors may be effective in treating DN. However, when there is excessive fructose intake, fructose metabolism causes energy loss that leads to uric acid production, causing inflammation and renal fibrosis. Renewal of existing beta cells is used in the treatment of DN. A novel insulin inhibitory receptor called an inceptor can be used for the treatment.
